# Interferon-γ regulates immunosuppression in septic mice by promoting the Warburg effect through the PI3K/AKT/mTOR pathway

**DOI:** 10.1186/s10020-023-00690-x

**Published:** 2023-07-11

**Authors:** Xu-zhe Fu, Yu Wang

**Affiliations:** Department of Emergency Medicine, Shengjing Hospital of China Medical University, Shenyang, China

**Keywords:** Sepsis, Interferon-gamma, Immunosuppression, Metabolism, Warburg effect

## Abstract

**Background:**

The main cause of high mortality from sepsis is that immunosuppression leads to life-threatening organ dysfunction, and reversing immunosuppression is key to sepsis treatment. Interferon γ (IFNγ) is a potential therapy for immunosuppression of sepsis, promoting glycolysis to restore metabolic defects in monocytes, but the mechanism of treatment is unclear.

**Methods:**

To explore the immunotherapeutic mechanism of IFNγ, this study linked the Warburg effect (aerobic glycolysis) to immunotherapy for sepsis and used cecal ligation perforation (CLP) and lipopolysaccharide (LPS) to stimulate dendritic cells (DC) to establish in vivo and in vitro sepsis models, Warburg effect inhibitors (2-DG) and PI3K pathway inhibitors (LY294002) were used to explore the mechanism by which IFNγ regulates immunosuppression in mice with sepsis through the Warburg effect.

**Results:**

IFNγ markedly inhibited the reduction in cytokine secretion from lipopolysaccharide (LPS)-stimulated splenocytes. IFNγ-treated mice had significantly increased the percentages of positive costimulatory receptor CD86 on Dendritic cells expressing and expression of splenic HLA-DR. IFNγ markedly reduced DC-cell apoptosis by upregulating the expression of Bcl-2 and downregulating the expression of Bax. CLP-induced formation of regulatory T cells in the spleen was abolished in IFNγ -treated mice. IFNγ treatment reduced the expression of autophagosomes in DC cells. IFNγ significant reduce the expression of Warburg effector-related proteins PDH, LDH, Glut1, and Glut4, and promote glucose consumption, lactic acid, and intracellular ATP production. After the use of 2-DG to suppress the Warburg effect, the therapeutic effect of IFNγ was suppressed, demonstrating that IFNγ reverses immunosuppression by promoting the Warburg effect. Moreover, IFNγ increased the expression of phosphoinositide 3-kinases (PI3K), protein kinase B (Akt), rapamycin target protein (mTOR), hypoxia-inducible factor-1 (HIF-1α), pyruvate dehydrogenase kinase (PDK1) protein, the use of 2-DG and LY294002 can inhibit the expression of the above proteins, LY294002 also inhibits the therapeutic effect of IFNγ.

**Conclusions:**

It was finally proved that IFNγ promoted the Warburg effect through the PI3K/Akt/mTOR pathway to reverse the immunosuppression caused by sepsis. This study elucidates the potential mechanism of the immunotherapeutic effect of IFNγ in sepsis, providing a new target for the treatment of sepsis.

## Background

Sepsis is a systemic disease with high mortality caused by pathogenic infection, affecting more than 49 million people worldwide yearly, with a mortality rate of approximately 26.7% (Rhee and Klompas 2020). According to epidemiological projections, this proportion will continue to increase due to increased bacterial resistance and an aging population (Rhee et al. 2019; Rudd et al. 2020). Aside from these, the challenges that sepsis poses to the healthcare system also include the proportion and cost of hospitalisation. The average length of hospital stay for patients with sepsis is approximately 75% longer than for patients of most other diseases. Sepsis management costs $41.5 billion annually in the United States alone, far exceeding the second most expensive disease, osteoarthritis (Paoli et al. 2018; Buchman, et al. 2020). The threat of sepsis is not just felt in developed countries but its effect is more severe in developing countries. For example, sepsis accounts for one-fifth of patients admitted to intensive care units (ICUs) in mainland China, with a 90-day mortality rate reaching as high as 35.5% (Fleischmann-Struzek et al. 2020). A recent European study of sepsis mortality showed regional differences, suggesting an unmet need for improved sepsis care (Bauer et al. 2020).

A new global consensus, Sepsis-3, defines sepsis as a life-threatening organ insufficiency resulting from a dysregulated host response to infection (Fernando et al. 2018). The main reason sepsis has such a high mortality rate is that when a body organ fails, the host enters into a state of immunosuppression or immune paralysis. This can be manifested as the apoptosis of immune effector cells, downregulation of major histocompatibility complex class II molecules, upregulation of negative co-stimulatory molecules, or the increase in the proportion of regulatory T cells (Tregs) and myeloid suppressor cells. Therefore, reversing immunosuppression in patients with sepsis at this stage is the focus of efforts to reduce mortality due to this disease.

Accumulating evidence has shown that activated immune cells, such as macrophages, dendritic cells (DC), and T cells, exhibit a phenomenon similar to the Warburg effect in tumour cells (Huang et al. 2018; O'Neill and Pearce 2016). In this condition, tumour cells rely on glycolysis to obtain ATP when the oxygen supply is normal. This suggests that the immune responses in sepsis and tumourigenesis may share similar metabolic shift mechanisms, contributing to the regulation of innate immune function. This brings new ideas for the immunotherapy of sepsis. At present, research on the treatment of sepsis through the Warburg effect mainly focuses on inhibiting the inflammatory response by inhibiting the Warburg effect. However, only a few studies focused on the relationship between the Warburg effect and immune function in sepsis. It has been found that the immune status of patients with sepsis changes with the development of the disease, and the weakening of the Warburg effect in these patients may be the key point of immune paralysis (Cheng et al. 2014a; Vught et al. 2016). Attenuation of the Warburg effect in patients with sepsis may be a key point in their immune paralysis. The mechanism underlying the Warburg effect mainly depends on the activation of the protein kinase B (Akt)-mTOR-hypoxia-inducible factor (HIF)-1α pathway (Pan et al. 2022a). Interfering with the Warburg effect in immune cells will affect their activation, differentiation, and memory formation.

Current immunostimulant drugs include, granulocyte–macrophage colony-stimulating factor (GM-CSF), IFNγ, interleukin 7 (IL-7) and interleukin 15 (IL-15), etc. GM-CSF can significantly improve infection clearance, but the expansion of pathologically activated MDSCs stimulated by GM-CSF cannot be solved, and IL-7 and IL-15 are designed to directly affect the T cell immunity of patients with sepsis.Our previous experiments found that interferon-gamma (IFNγ)-targeted immune enhancement can treat sepsis (Darden et al. 2021). IFNγ can increase cytokine production, reduce T cell apoptosis, and decrease the proportion of regulatory T cells. IFNγ also reduces CD4 + T cell inhibitory receptor expression and systemic inflammation in septic mice; however, the mechanism by which IFNγ exerts immune regulation in sepsis is unclear (Wang et al. 2017). Studies have shown that IFNγ treatment can promote glycolysis to restore the function of monocytes in the peripheral blood of patients with sepsis (Cheng et al. 2016). Here, we hypothesise that IFNγ promotes the Warburg effect through the PI3K/Akt/mTOR pathway in treating septic mice via immunosuppression. We used caecal ligation and puncture (CLP) and lipopolysaccharide (LPS) stimulation to induce a sepsis model, using key pathways (PI3K/AKT/MTOR) and Warburg effect-related indicators (Ratajczak-Wrona et al. 2014; Wrann et al. 2007). Moreover, we also used glycolysis inhibitors (2-deoxy-d-glucose, 2-DG) and a PI3K inhibitor (LY294002) to investigate whether IFNγ reverses sepsis-induced immunosuppression by promoting the Warburg effect (Wijayasinghe et al. 2022). 2-DG is a glucose analogue that is a competitive glycolysis inhibitor and has no effect on immune function and LY294002 is a highly selective inhibitor of PI3K (Pajak et al. 2019; Vlahos et al. 1994).

In this study, the CLP method was used to replicate a sepsis mouse model, and a septic DC culture system was constructed. We then determined the immunoregulatory effect of this method on septic mice and the Warburg effect on immune cells to explore the molecular mechanism underlying the IFNγ-mediated regulation of immune suppression. Explain the immunostimulation therapy mechanism of IFNγ, find new targets for immunostimulation therapy for sepsis, and enrich the individualized treatment of sepsis.

## Methods

### Animals

In this study, 6- to 8-week-old SPF-grade male C57BL/6 mice (weight 25–30 g) were purchased from the Experimental Animal Center, Chinese Academy of Medical Sciences. Animals were acclimated to random food and water for 1 week before the experiment. The mice were kept in an environment with a temperature of 20–25 ℃, humidity of 40–70%, and a 12:12 h light/dark cycle. All studies were performed at the same time of day to avoid circadian rhythm effects. All animal experiments were reviewed and approved by the Ethics Committee of Shengjing Hospital Affiliated with China Medical University, ethics code: 2021PS458K.

### Establishment of a mouse CLP model

The sepsis mouse model was established using the CLP method (Dejager et al. 2011). Briefly, mice were anaesthetised via inhalation with 2% isoflurane. The mice were then placed on mouse plates, fixed, shaved, and sterilised thrice. A longitudinal incision was made in the middle of the lower abdomen along the linea alba to free the caecum. Then, 5–0 sutures were used to ligate 50% of the caecum (50% of the total length of the caecum from the free end to the ligation site, mid-grade sepsis). At the midpoint between the blind end and the ligation site, a 22-gauge needle was used to penetrate the caecum from the avascular side of the mesentery to the non-mesenteric side, squeezed out 2 mm of feces, and the abdomen was closed. Resuscitation fluid (normal saline, 50 mL/kg) and analgesic (buprenorphine, 0.05–0.1 mg/kg) were administered via subcutaneous injection. The experimental mice in the sham-operated group underwent only laparotomy incision without cecum ligation and perforation steps, while all other steps were the same as in the sepsis group.

### Experimental animals

Eighty-four male C57BL/6 mice were randomly divided into seven groups (n = 12 animals in each group): sham operation group, CLP group (sepsis group), IFNγ group, CLP + IFNγ group, 2-DG group, 2-DG + CLP group, and IFNγ + 2-DG + CLP group. Thirty minutes before establishing the CLP-induced sepsis model, IFNγ was administered as recombinant human interferon (0.01 μg/g body weight, Peprotech, NJ, USA) diluted to 0.5 mL in normal saline and injected intravenously through the tail vein. The control group was given the same amount of normal saline. Mice in the 2-DG group were intraperitoneally injected with 2-DG (500 mg/kg, APExBIO, Houston, TX, USA) diluted to 0.5 mL with normal saline 3 h before establishing the CLP-induced sepsis model. The control group was given the same amount of normal saline. After 24 h of molding, the mice were sacrificed by CO_2_, and serum and spleen samples were collected for further analysis.

### Cell culture

DC2.4 cells (Procell, Wuhan, China) were selected and cultured in RPMI1640 medium (Solarbio, Beijing, China) supplemented with l-glutamine (2 mM), streptomycin (100 μg/mL), penicillin (100 U/mL), non-essential amino acids (100 μM), 2-mercaptoethanol (2-ME, 50 μM), and 10% foetal bovine serum (FBS, Procell) in an incubator (37 °C, 5% CO_2_). These cells were divided into eight groups: the normal group (control group), CLP group (LPS, 5 µg/mL), IFNγ group (20 ng/mL IFNγ), IFNγ + CLP group, 2-DG group (25 mM 2-DG), 2-DG + CLP group, IFNγ + 2-DG + CLP group, and LY294002 + IFNγ + CLP group (LY294002, 10 mM). Each group was incubated with the respective treatments for 24 h, after which the cells and supernatants were collected.

### Cell viability detection using the CCK-8 assay

DC2.4 cells were trypsinised to prepare suspensions with a cell density of 10^4^ cells/100 µL. Next, 100 µL cell suspensions were placed into each well of a 96-well plate and cultured in an incubator for 24 h. Thereafter, 10 µL of different concentrations of each test substance (100 ng/mL, 200 ng/mL, 400 ng/mL, 800 ng/mL, 1 µg/mL, 5 µg/mL, 10 µg/mL, 20 µg/mL, and 50 µg/mL) were added to each well, including the cell-only control (without the test substances) and medium-only control (without cells). After 24 h of incubation with the test drugs, 10 µL of CCK-8 solution (seven biotech, Beijing, China) was added to each well. A microplate reader was used to detect the cell viability under stimulation with different LPS concentrations, as well as the cell viability of each treatment group.

### Histopathological analysis of spleen tissue

The mice were sacrificed after anaesthesia, and their spleen tissues were fixed in 4% paraformaldehyde and sliced into tissue blocks with thicknesses of approximately 0.5 cm. Routine gradient alcohol dehydration, paraffin embedding, serial sectioning, and haematoxylin and eosin (HE) staining were then performed. The histopathological changes in spleen tissue were observed under a light microscope. Spleen histopathological analysis was performed using a semi-quantitative scoring system, which includes the area of B and T lymphocytes in red and white pulp (0, absent; 1, mild; 2, moderate; 3, marked) and the presence of apoptotic cells, macrophages, necrotic cells, and pigments (0, absent; 1, present). Segments of the spleen were scored (Badr et al. 2011).

### Primary cell isolation and culture

Mice were killed after anaesthesia and immersed in 70% alcohol for 5 min. Their spleens were harvested under sterile conditions, and the spleen capsules were torn off. A 200-mesh filter was then placed on a 60-mm dish, and a small amount ofPBS was added. The spleen was placed on the filter and was ground using the handle of a syringe. After grinding, the filter and syringe handle were rinsed with tissue diluent to collect the cell suspension. The cell suspension was centrifuged (1400 rpm, 4 °C, 10 min), and the supernatant was discarded. Next, 5 mL phosphate-buffered saline (PBS) was added to the pellet, mixed well, then centrifuged again with the same parameters. The supernatant was discarded, 2 mL of red blood cell lysate was added to the pellet, and the cells were mixed. The cell suspension was then placed on ice for 15 min, shaking every 5 min. Thereafter, the cell suspension was centrifuged (1400 rpm, 4 °C, 10 min), and the supernatant was discarded. The final cell pellet was then washed with PBS and resuspended in 15 mL of RPMI1640 medium supplemented with 10% FBS. Finally, the cells were cultured in a T75 culture flask and placed in an incubator.

### Flow cytometric analysis of spleen cells

Splenocyte suspensions were incubated with isotype controls or specific antibodies, and differences were reduced with normal cells and single staining. Splenocytes were stained with APC-labelled anti-CD11c, FITC-labelled CD86, and PE-labelled PD-L1 antibodies (Biolegend, San Diego, CA, USA). Labelled cells were then separated using a FACSCalibur flow cytometer and CellQuest software (BD Biosciences, Pharminogen), and data were analysed with FlowJo 10.8.1 software.

### Regulatory T cell analysis

Splenocyte isotype controls or specific antibodies were incubated with normal cells and single staining was performed to reduce differences. Splenocytes were stained with APC-labelled anti-CD4, FITC-labelled CD25, and PE-labelled Foxp3 antibodies (Biolegend). First of all, CD4, CD25 room temperature away from light staining for 30 min, Foxp3 staining before using a film breaker (Elabscience), according to the instructions, Foxp3 room temperature away from light staining for 30 min. Labelled cells were analysed using a FACSCalibur flow cytometer and CellQuest software (BD Biosciences), and the data were analysed with FlowJo 10.8.1 software.

### Splenocyte cytokine production

Mononuclear cells were prepared via density gradient centrifugation (Solarbio). The cells were then stained with trypan blue (Solarbio) to detect cell viability > 85%. The isolated mononuclear cells were stimulated with LPS (10 ng/mL; Solarbio). After 6 h, the cell supernatant was collected, and an enzyme-linked immunosorbent assay (ELISA, Elabscience, Wuhan, China) was used to detect the levels of the cytokines tumour necrosis factor (TNF)-α, interleukin (IL)-6, and IL-10, in strict accordance with the kit instructions.

### Detection of plasma TNF-α, IL-6, and IL-10 levels

Blood was collected from the inferior vena cava (1:10 acid dexamethasone citrate) and centrifuged at 10,000 × *g* for 10 min. The supernatant was then collected and stored at − 80 °C until use. Plasma levels of TNF-α, IL-6, and IL-10 were detected using ELISA kits (Elabscience) according to the manufacturer’s instructions.

### Immunohistochemical detection of HLA-DR and Caspase-3

The obtained spleen tissue was fixed in 4% paraformaldehyde at 4 ℃ for 48 h and embedded in paraffin. The paraffinised sections (0.3 μm) were dewaxed with water, and the antigens were heat-recovered at a high temperature. The sections were then blocked with H_2_O_2_ for 40 min at room temperature, washed with PBS, and then blocked with serum (Elabscience) for 40 min at room temperature. Paraffin sections were incubated overnight at 4 °C with antibodies against HLA-DR (BOSTER, CA, USA) and Caspase-3 (Cell Signaling Technology, Boston, MA, USA). The sections were then washed thoroughly with PBS and then incubated with the corresponding biotin-labelled secondary antibodies (Elabscience) for 20 min at room temperature at 37 °C. Thereafter, the sections were incubated with diaminobenzidine (DAB, Elabscience), developed, and counterstained with haematoxylin. The negative control was incubated with PBS instead of the secondary antibody. Brown-yellow or brown-yellow particles with a ring-shaped distribution indicate positive results. Images were captured using an NIS-Elements F2.30 image acquisition software (Laboratory Imaging, Hostivař, Czech Republic). The average optical densities (ODs) of the positive areas were measured with ImagePro Plus software (Media Cybernetics, Rockville, MD, USA), reflecting the expression level of the corresponding products.

### Western blot analysis

For western blot analysis, 100 µL of each sample (20 mg spleen tissue or 6 × 10^6^ cells) was dissolved in high-strength RIPA buffer (Epizyme, Shanghai, China) supplemented with PMSF (protease inhibitor, RIPA:PMSF = 100:1) and PI (phosphatase inhibitor, RIPA:PI = 100:1). The samples were ultrasonicated on ice for 30 min and then centrifuged to collect the supernatant, 12000x*g*, 30 min, 4 °C. A BCA assay (Vazyme, Nanjing, China) was used to determine the protein concentration, and RIPA was added to adjust the sample concentration. Other samples were dissolved in SDS-PAGE protein loading buffer (Beyotime, Shanghai, China), boiled at 100 °C for 10 min, and then stored at − 80 °C. The protein samples were then separated using a PAGE Gel Fast Preparation Kit (Epizyme) and then transferred to polyvinylidene fluoride membranes. Protein sizes were determined using a two-colour pre-stained protein marker (Epizyme) at the recommended concentration of 4 °C. The membranes were then incubated overnight with primary antibodies (anti-LDH, 1:1000; anti-PDH, 1:1000; anti-GLUT1, 1:1000; anti-GLUT4, 1:500; and anti-HIF-1α, 1:1000; Affinity Biosciences, USA; anti-AKT, 1:1000; anti-p-AKT, 1:1000; Cell Signaling Technology; anti-PI3K, 1:1000; anti-p-PI3K, 1:1000; anti-mTOR, 1:2000; anti-p-mTOR, 1:2000; and anti-PDK1, 1:1000; Abcam, Cambridge, UK). After washing with TBST, the membranes were incubated with the corresponding secondary antibodies (1:4000, Elabscience) at room temperature. Chemiluminescence development (ECL; Vazyme) was performed after washing the membranes with TBST. ImageJ software (National Institutes of Health, Bethesda, MD, USA) was used for analysis.

### Quantitative reverse transcription polymerase chain reaction (qRT-PCR)

Total RNA was extracted from splenocytes using an RNAkey RNA extraction kit (Seven Biotech, Beijing, China) following the manufacturer’s protocol. cDNA was synthesised from the total RNA using a PrimeScript™ RT Kit with gDNA Eraser Reverse Transcription System (Takara, Japan). Real-time polymerase chain reaction (RT-PCR) was performed using a TB Green® Premix Ex Taq™ II (Takara) in an Applied Biosystems 7500 system with Applied Biosystems 7500 Plus software (Applied Biosystems, Waltham, MA, USA). The oligonucleotide primer sequences used were as follows: Bcl-2 (156 bp), forward primer: 5*'*-GATGACTTCTCTCGTCGCTAC-3*'*, reverse primer: 5*'*-GAACTCAAAGAAGGCCACAATC-3*'*; Bax (140 bp), forward primer: 5*-*GCTACAGGGTTTCATCCAGGATC-3*'*, and reverse primer: 5*'*-TGCTGTCCAGTTCATCTCCAATTCG-3*'*; and β-actin (203 bp), forward primer: 5*'*-TCCTTCTTGGGTATGGAAT-3*',* and reverse primer: 5*'*-GAGCAATGATTTGATTC-3*'*. Each 20 μL qRT-PCR mix contained 10 μL TB Green® Premix Ex Taq™ II (Takara), 0.4 μL ROX Reference Dye II. 0.8 μL of each primer, and 2 μL cDNA as template, adjusting the volume to 20 μL with sterile enzyme-free water. The qRT-PCR conditions were as follows: denaturation at 95 °C for 10 min, followed by 40 cycles of denaturation at 95 °C for 30 s, annealing at 55 °C for 1 min, and extension at 72 °C for 1 min. The relative differences in the expression of Bcl-2 and Bax between groups were determined using cycle threshold (Ct) values.

### Metabolic analysis

Glucose consumption was determined by detecting glucose content using a colorimetric method (Elabscience). Animal experiments use primary cells of the spleen. An L-Lactic Acid/Lactate (LA) Colorimetric Assay Kit was used to detect lactate production in the serum and DC2.4 cell culture supernatants. An ATP Colorimetric Assay Kit was used to detect the amount of ATP generated in the tissue samples. An Adenosine Triphosphate (ATP) Chemiluminescence Assay Kit was used to detect intracellular ATP production. All kits used in the assays mention in this subsection were purchased from Elabscience and were performed in accordance with the respective product manuals.

### Terminal deoxynucleotidyl transferase dUTP nick-end labelling (TUNEL) assay

5 × 10^4^DC2.4 cellswere plated on 24-well plate with chamber slides. The cells were washed twice with PBS, fixed with 4% paraformaldehyde (4 °C, 30 min), washed with PBS, and then treated with pre-cooled 70% ethanol (− 20 °C, 4 h). The cells were then washed with PBS, 100 µL of TUNEL equilibration buffer was added, and the cells were incubated for 5 min. The buffer was discarded, the excess liquid was blotted off with filter paper, and 50 µL TUNEL reaction solution was added. The samples were then covered with buffer, and then incubated at 37 °C for 60 min. The reaction solution was removed and the cells were washed with PBS for 5 min. The cells were then permeabilised using 0.1% Triton X-100 (containing 5 mg/mL BSA) in PBS, washing thrice for 5 min each time in PBS. DAPI counterstaining. Dropped onto adhesive slides and covered with cell slides. Finally, images were captured using NIS-Elements F2.30 image acquisition software (Laboratory Imaging). Statistical analyses were performed using ImagePro Plus software (Media Cybernetics).

### Detection of autophagosomes via confocal microscopy

Briefly, the DCs were collected, and the number of intracellular autophagosomes (characterised by fluorescent antibodies bound to the LC3 puncta of autophagosomes) was observed via laser confocal microscopy to determine the changes in the autophagic activity of DCs in different groups. The harvested DCs (approximately 2 × 10^6^ cells) were transferred to flow tubes. Next, 400 µL of 0.025% digitonin (250 µg/mL, Merck, Darmstadt, Germany) was added to each flow tube, and the membrane was broken for 2 min on ice. Cells were resuspended in 1 mL PBS, washed three times via centrifugation (1500 rpm, 5 min), and then fixed with 4% paraformaldehyde for 1 h at room temperature. The cells were then blocked with 1% BSA blocking solution for 1 h at room temperature. Next, the cell suspensions were centrifuged, washed twice with PBS, and 200 µL anti-LC3B primary antibody (1:200, Cell Signaling Technology) was added. The cells were incubated overnight at 4 °C, washed twice via centrifugation in PBS, and 200 µL of the corresponding fluorescently labelled secondary antibodies were added (1:200). Next, the cells were incubated for 1 h at room temperature in the dark, centrifuged, and then washed thrice with PBS. Forty microliters of each cell suspension were then mixed with 10 µL DAPI staining solution, dropped onto adhesive glass slides, and the autophagosomes were observed under a Leica SP8 confocal microscope.

### Statistical analysis

Data are presented as the mean ± SEM. Statistical analyses were performed using one-way analysis of variance followed by a multiple comparisons test (Student–Newman–Keuls method). Differences were considered significant at p < 0.05. ns: p > 0.05, *p < 0.05, **p < 0.01, vs. con/sham;. #p < 0.05, ##p < 0.01, vs. CLP/LPS; &p < 0.05, &&p < 0.01, vs. IFNγ + CLP/LPS.

## Results

### IFNγ treatment can restore the immune function of septic mice; 2-DG can inhibit the therapeutic effect of IFNγ

#### General observation of experimental animals

**Fig. 1:**
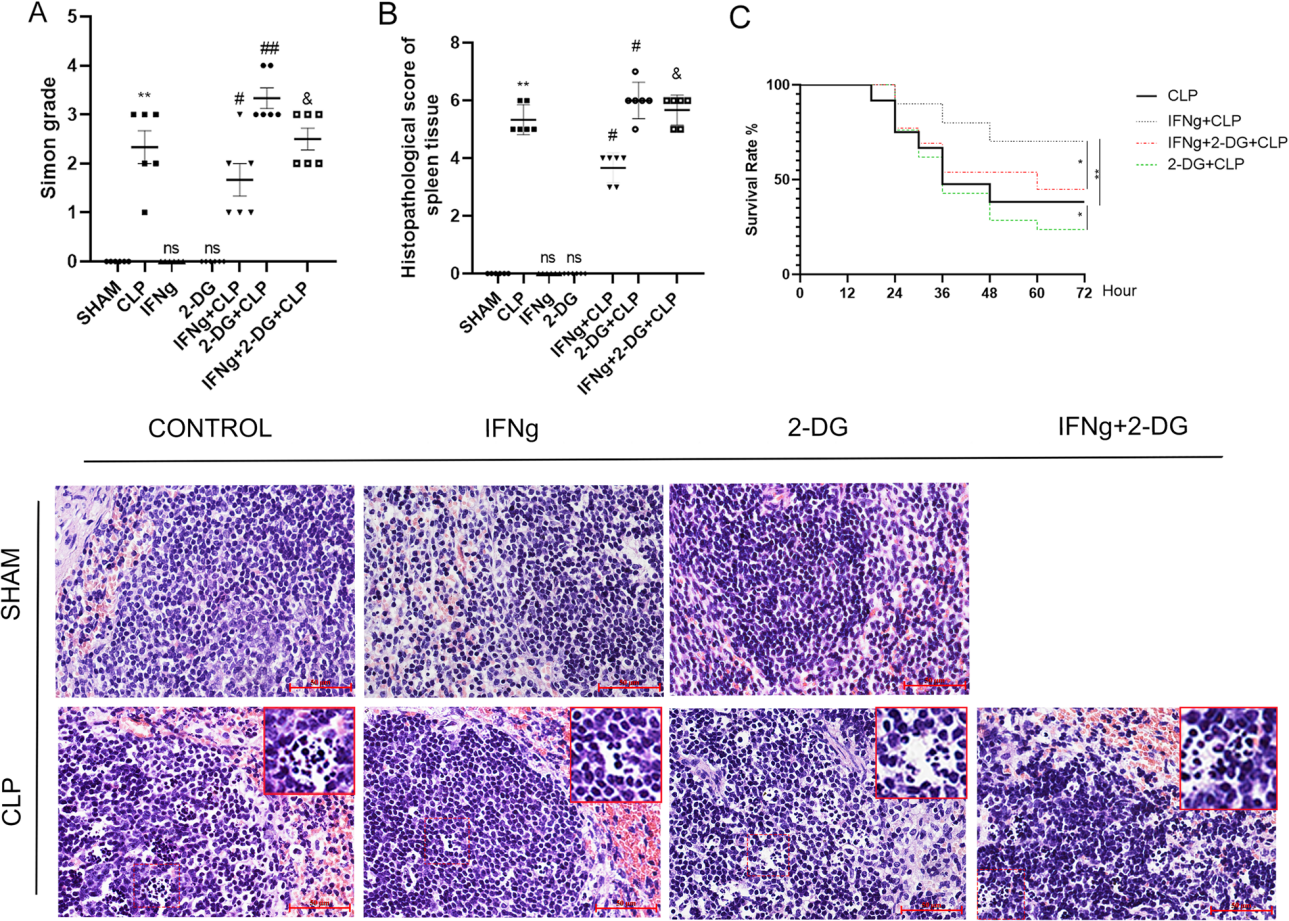
**A** Simon grade and **B** Histopathological changes (Hematoxylin–eosin staining) in the spleen tissue of mice in the seven treatment groups. **C** Survival rate.The scale bar represents 50 μm. Data are expressed as mean ± SEM (n = 3). Significance levels are indicated as follows: ns, not significant; *p < 0.05; #p < 0.05; &p < 0.05

We observed that the mice in the sham-operated group, IFNγ group, and 2-DG group woke up quickly after modelling. They quickly returned to normal drinking and eating, and their activities were normal. After surgery, the mice in the CLP group began to have decreased food intake, decreased activity, increased respiratory rate, lethargy, piloerection, chills, increased secretions at the corners of the mouth and eyes, and weakened protective reflexes. As their disease progressed, the above symptoms worsened; haematuria, pyuria, and diarrhoea also became present. Murine autopsies revealed massive, foul-smelling, bloody exudates in the abdominal cavity, flatulence of the small intestine, decreased peristalsis, swelling, necrosis and adhesion of the caecum at the ligated end, congestion, and oedema of other abdominal organs. The performance of mice in the IFNγ + 2-DG + CLP group was similar to that in the CLP group. Mice in the IFNγ + CLP group showed only mild shortness of breath, decreased activity, lethargy, decreased intraperitoneal exudation, presence of small abscesses formed by adhesion of the caecal ligation site, and only a mild dilation of the intestinal tract. Compared with the sepsis group, the mice in the 2-DG + CLP group had more severe peritoneal blood exudation, caecal swelling, and necrosis at the ligated end of the caecum. According to the Simon classification(Taşcı et al. 2017), the mice in the 2-DG + CLP group had grade 3–4 abdominal infection, the IFNγ + 2-DG + CLP and CLP groups were at grade 2–3, the IFNγ + CLP group was at grade 1–2, and the sham group was at grade 0. The surgery, IFNγ, and 2-DG groups were at grade 0 (Fig. 1A). Comparing the survival rate of CLP group with IFNg + CLP group, IFNg can improve the survival rate of mice with sepsis and 2-DG decreases the survival rate of mice (Fig. 1C).

#### Histopathological analysis of the spleen

According to the pathological score, HE staining of spleen tissue in the sham, IFNγ, and 2-DG groups showed no significant difference. In the CLP group, the splenic sinus was dilated, the membrane was thickened, infiltration of neutrophils was obvious, the number of splenic nodules was increased, and the germinal centre was obvious. Compared with the CLP group, the spleens of mice in the 2-DG + CLP group were severely damaged and the number of apoptotic cells was increased. Compared with the IFNγ + CLP group, the spleens of mice in the IFNγ + 2-DG + CLP group had more severe damage (Fig. 1B).

#### Analysis of immune function

**Fig. 2:**
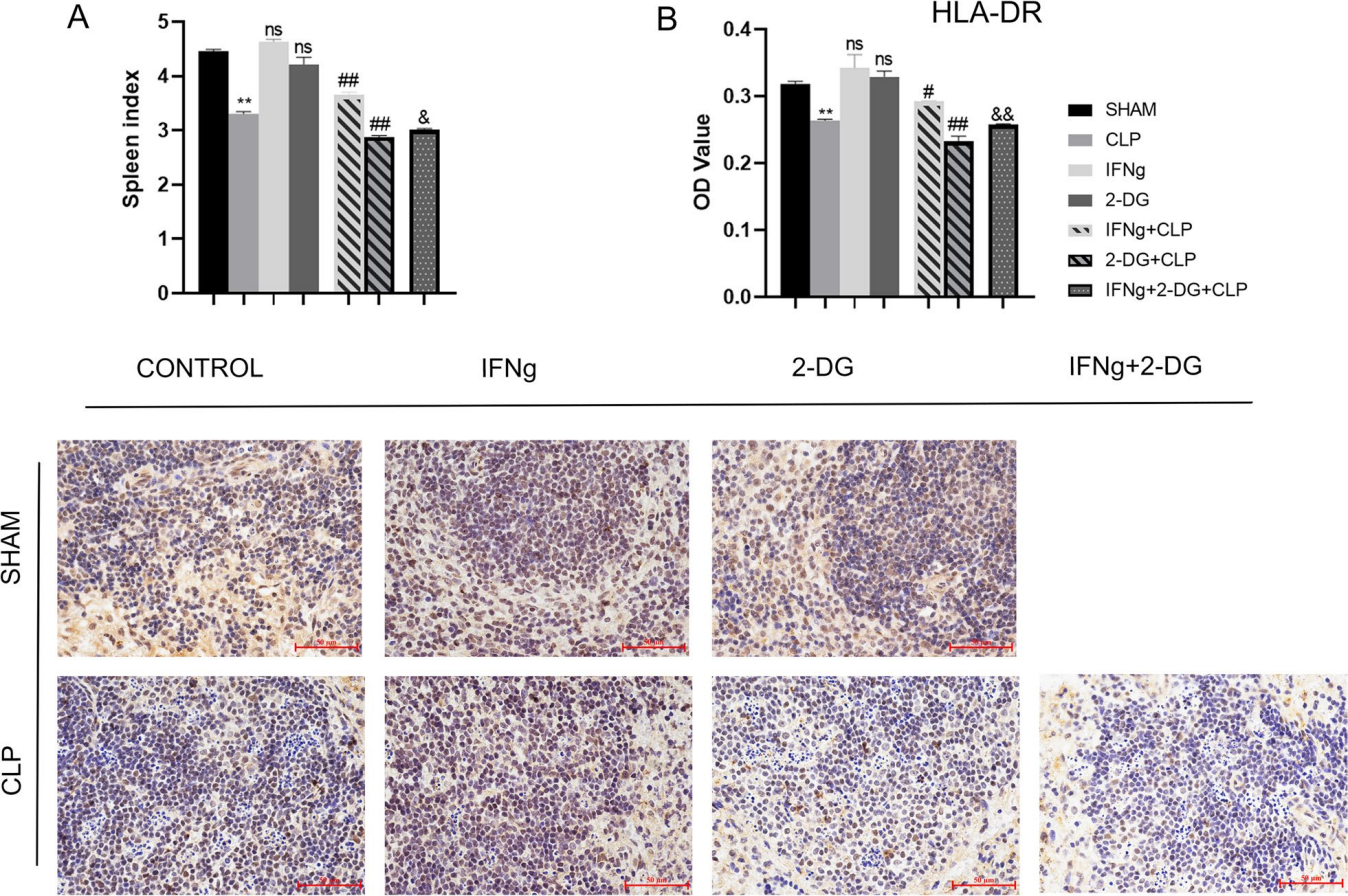
**A** Spleen index of the seven groups of mice. **B** Immunohistochemical staining for HLA-DR. Scale bars represent 50 μm. Data are expressed as the mean ± SEM (n = 3). Significance levels are indicated as follows: ns, not significant; *p < 0.05, **p < 0.01, #p < 0.05, ##p < 0.01, &p < 0.05, &&p < 0.01

**Fig. 3:**
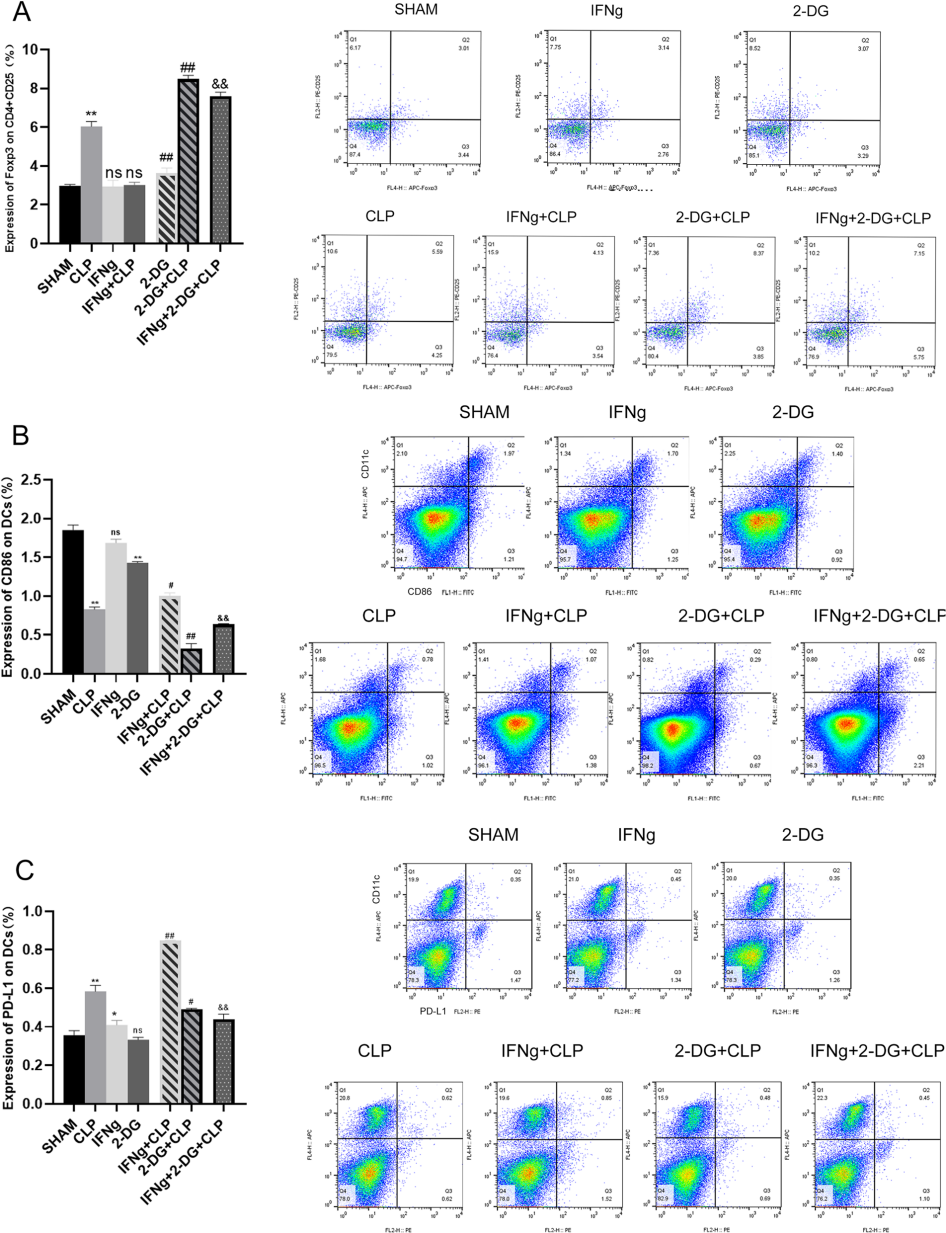
**A** The percentage of regulatory T cells (CD4^+^ CD25^+^ Foxp3^+^) in the spleen of mice 24 h after CLP induction was detected by flow cytometry. **B** CD86 expression on the DC surface as detected via flow cytometry. **C** PD-L1 expression on the DC surface as detected via flow cytometry. Data are expressed as the mean ± SEM (n = 3). Significance levels are indicated as follows: ns, not significant; *p < 0.05, **p < 0.01, #p < 0.05, ##p < 0.01, &p < 0.05, &&p < 0.01

It can be seen from the animal model that CLP resulted in severe infection lesions, and the immune function of the spleen was also affected. In order to better study the immune function of mice in this study, changes in immune function were detected from different angles using multiple immune indicators, such as the spleen index, spleen HLA-DR expression, Treg ratio, and expression of CD86 and PD-L1 on the surface of immune DCs. The spleen index (spleen weight [mg]/mouse weight [g]) can roughly reflect the immune function of the spleen. HLA-DR is expressed on antigen-presenting cells. It presents extracellular proteins, plays a central role in the immune system, and can be used to monitor the impact on immunity (Zhuang et al. 2017). The brown-yellow colour is characteristic of DAB staining, and the nuclei are stained with haematoxylin. CD4^+^CD25^+^Foxp3^+^ Tregs represent a natural Treg subset that mainly exerts an immunosuppressive effect through cell contact (Chen et al. 2021). CD86 is an immunoglobulin expressed by antigen-presenting cells and is involved in T lymphocyte proliferation and activation (Du et al. 2022). PD-L1 is a negative regulator of T and B cells and plays an important role in mediating lymphocyte tolerance to self-antigens (Chen et al. 2021). Our results showed that CLP led to a significant decrease in the spleen index, decrease in HLA-DR and CD86 expression on the DC surface, an increase in Treg percentage and PD-L1 on the DC surface, and a significant decrease in spleen immune function. IFNγ treatment can restore immune function, improve the spleen index, increase HLA-DR and CD86 expression on the DC surface, and decrease Treg percentage, but IFNγ promotes PD-L1 expression. Treatment with 2-DG significant aggravate the immune function damage induced by CLP, increase the spleen index, decrease the expression of CD86 and HLA-DR on the DC surface, and increase the percentage of Tregs, but inhibit the expression of PD-L1. Moreover, the results have shown that 2-DG + CLP and IFNγ + 2-DG + CLP significant inhibited the immunotherapeutic effect of IFNγ (Figs. 2A, B, 3A–C).

#### Cytokine analysis in mice with sepsis

**Fig. 4:**
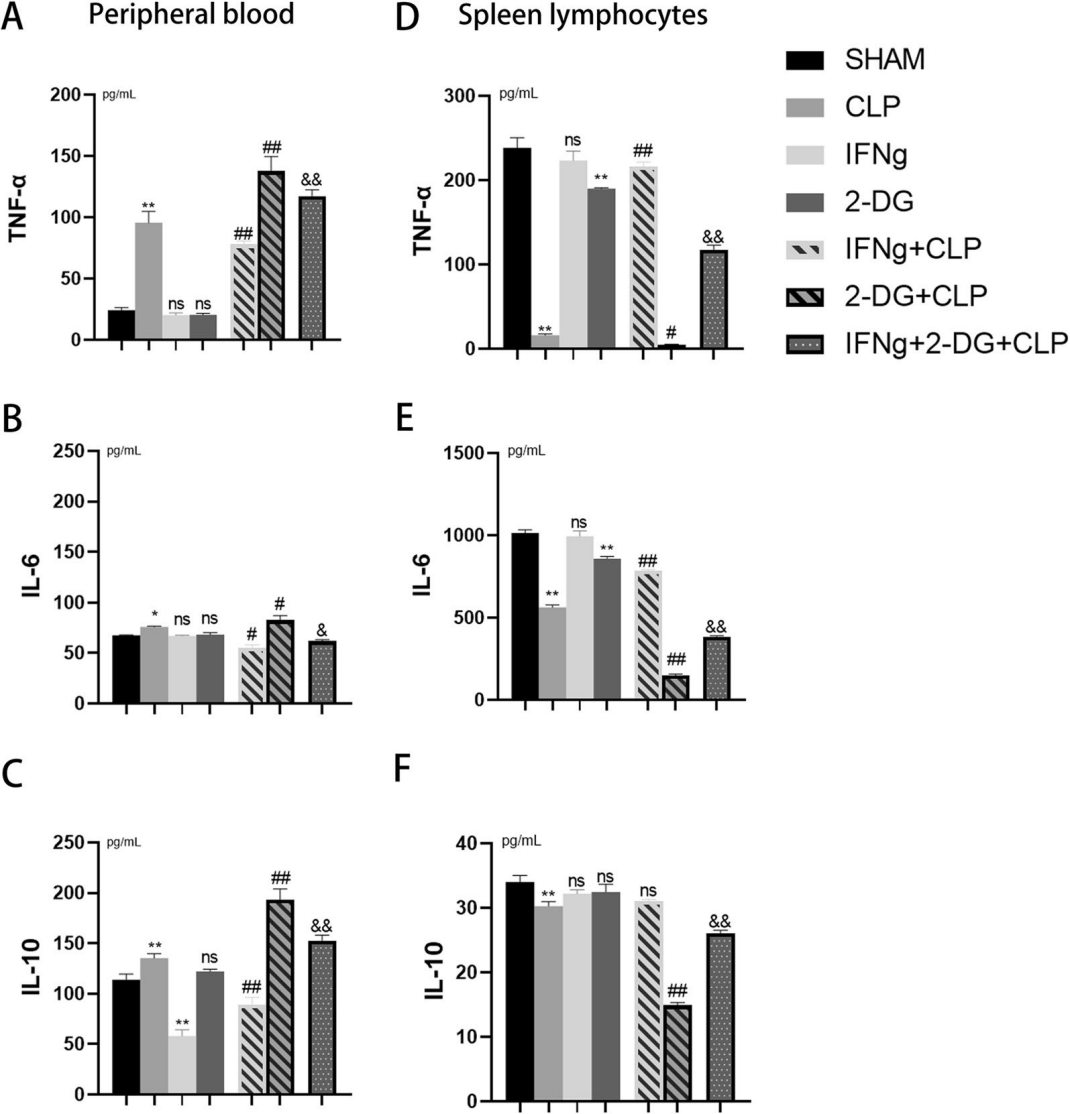
**A**–**C** Levels of TNF-α (**A**), IL-6 (**B**), and IL-10 (**C**) in plasma were detected using double-antibody sandwich enzyme-linked immunosorbent assay (ELISA) 24 h after CLP induction. **D**–**F** Double-antibody sandwich ELISA to detect the cytokines TNF-α (**D**), IL-6 (**E**), and IL-10 (**F**) in the mouse primary cells of each group after 6 h of LPS stimulation. Data are expressed as the mean ± SEM (n = 6). Significance levels are indicated as follows: ns, not significant; *p < 0.05, **p < 0.01, #p < 0.05, ##p < 0.01, &p < 0.05, &&p < 0.01

ELISA-based detection of the levels of TNF-α, IL-6, and IL-10 in the peripheral blood of mice 24 h after CLP induction revealed that cytokine content in the peripheral blood increased during the development of sepsis **(**Fig. 4A–C**)**. Since the cytokine content in peripheral blood is the accumulated amount secreted at any time point, the secretory ability of immune cells only in the immunosuppressive stage cannot be confirmed. In this study, we performed primary cell culture, wherein seven groups of mice were modelled. Primary cells were extracted 24 h later, and the primary cells from each group were stimulated with LPS. The secretion of cytokines (TNF-α and IL-6) decreased while the secretion of the negative regulator IL-10 increased after 6 h of LPS stimulation in primary cells of septic mice. It has been proven that the immune system releases a large number of cytokines in response to sepsis and that these cytokines are distributed throughout the body with the peripheral blood. However, 24 h after the induction of sepsis, with the development of the disease, CLP mice entered a period of immunosuppression, and the secretion of cytokines, whether pro- or anti-inflammatory, in the immune cells of these mice was inhibited **(**Fig. 3D–F**)**. From the peripheral blood or primary cell culture supernatants, it can be seen that IFNγ restored the secretion capacity of immune cell cytokines TNF-α and IL-6 but did not promote the secretion of the negative regulator IL-10. The effect of IFNγ on lymphocyte function is not only limited to the massive secretion of cytokines in the stage with a strong inflammatory response; it also can restore the cytokine secretion function of lymphocytes during the immunosuppression stage. In contrast, 2-DG inhibited the secretory function of lymphocytes. Moreover, after 2-DG pretreatment, the ability of IFNγ to restore the cytokine secretion of CLP mice was also inhibited.

### IFNγ treatment can inhibit sepsis-induced apoptosis; 2-DG and LY294002 can inhibit the therapeutic effect of IFNγ

**Fig. 5:**
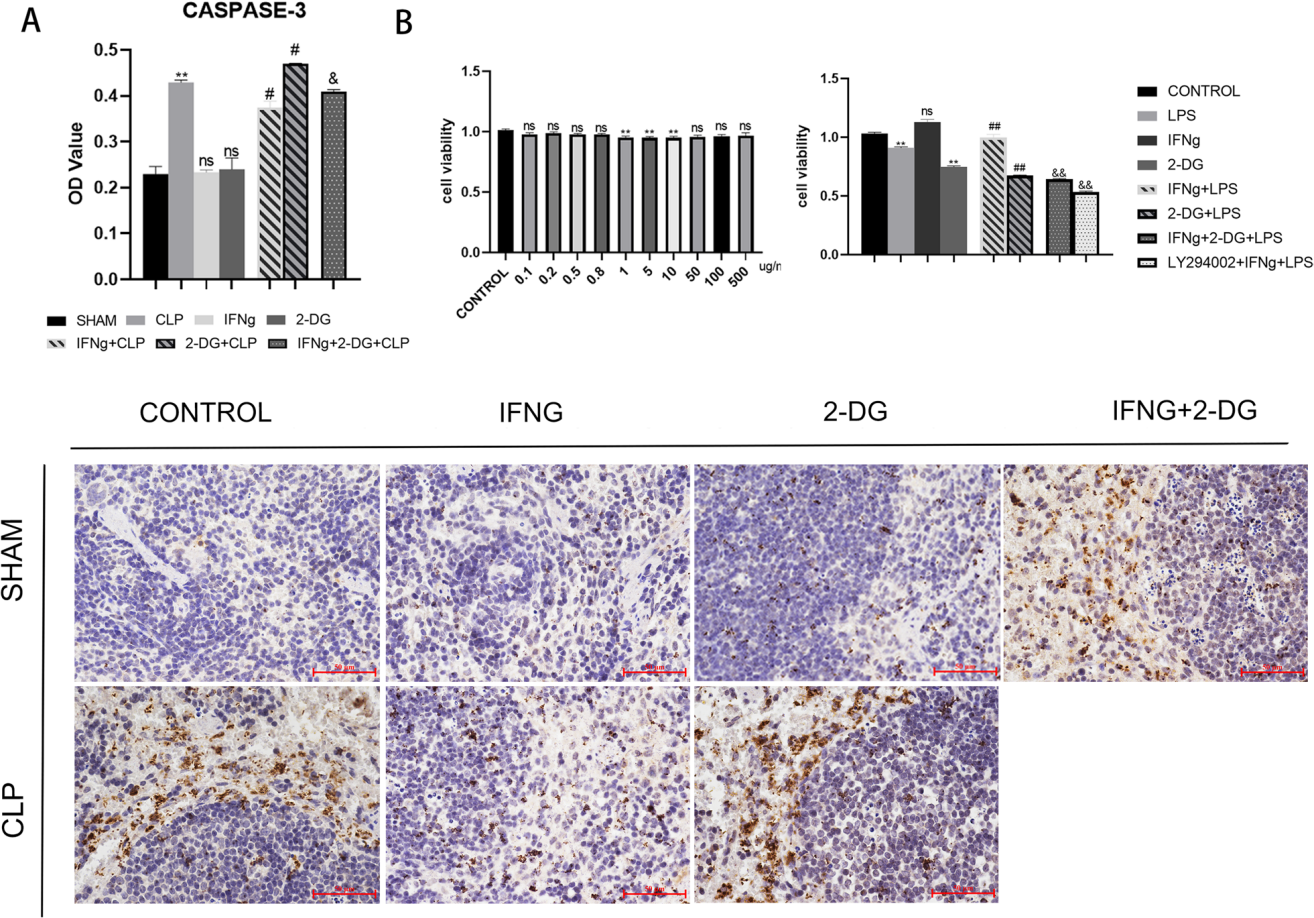
**A** Immunohistochemical staining for mouse caspase-3. The target cells were stained with DAB (brown) as a chromogenic reagent and were counterstained with haematoxylin (blue) for background staining. Caspase-3 immunohistochemical staining magnification: × 400. Data are expressed as the mean ± SEM (n = 3). **B** A CCK8 assay was used to detect the viability of DC2.4 cells, recording the absorbance at 450 nm using a microplate reader. Data are expressed as the mean ± SEM (n = 6). Significance levels are indicated as follows: ns, not significant; *p < 0.05, **p < 0.01, #p < 0.05, ##p < 0.01, &p < 0.05, &&p < 0.01

Caspase-3 is the most important terminal cleavage enzyme during apoptosis. It is highly expressed in immune cells and is typically localised in the cytoplasm. From the HE staining and Caspase-3 immunohistochemical staining, it can be seen that CLP can significantly damage the spleen and that IFNγ treatment can reverse this effect to some extent. These data demonstrate that IFNγ treatment can reverse the sepsis-induced damage in mice and that 2-DG can inhibit the therapeutic effect of IFNγ (Fig. 5A).

**Fig. 6:**
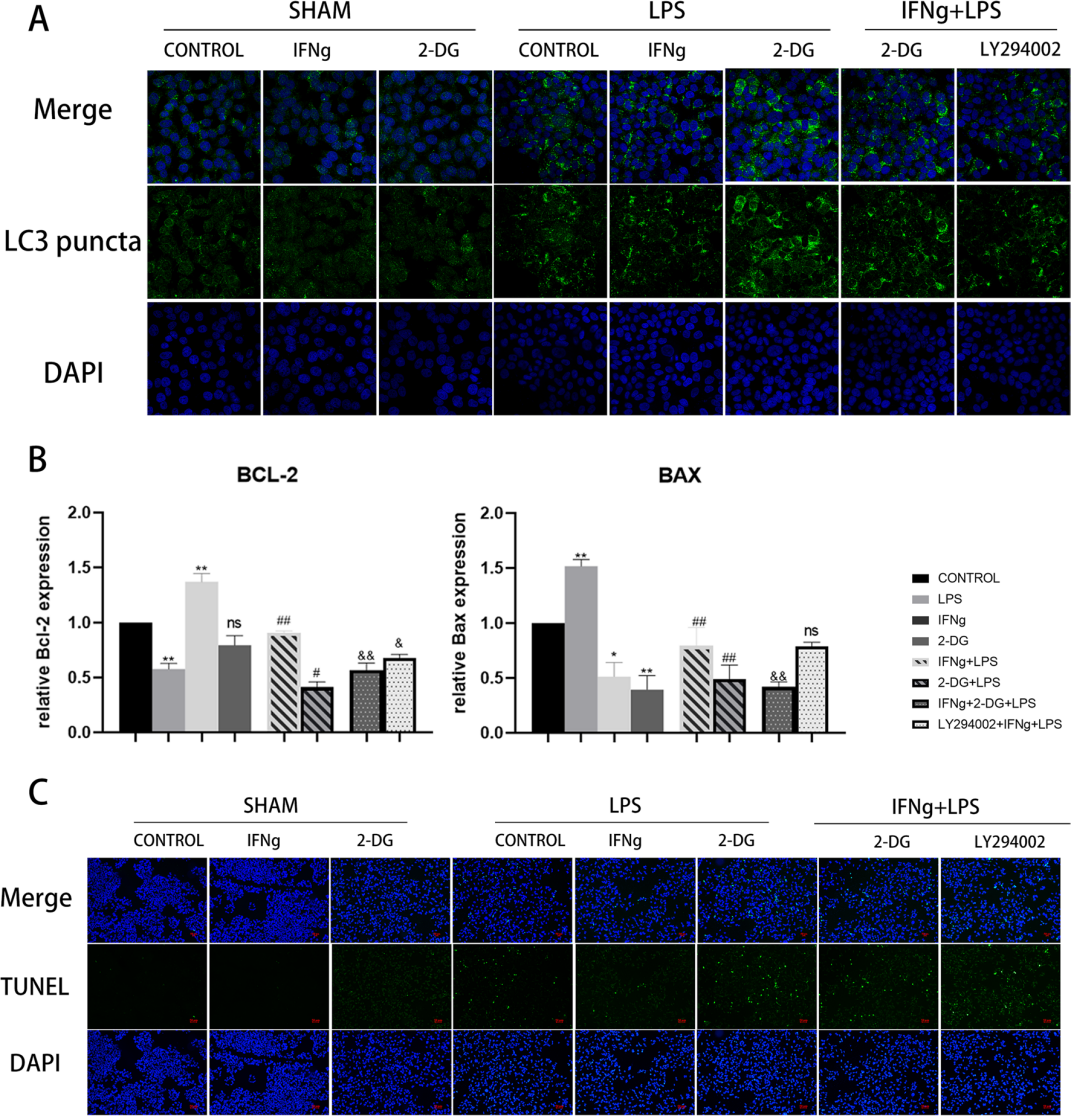
**A** LC3 puncta marked the autophagosomes, showing that LPS-induced sepsis increased autophagosome expression. IFNγ treatment decreased sepsis autophagosome expression. Treatment with 2-DG increased autophagosome expression; hence, 2-DG and LY294002 inhibited the therapeutic effect of IFNγ. **B** The expression levels of Bcl-2 and Bax mRNA in splenocytes were detected via quantitative reverse transcription polymerase chain reaction. Data are expressed as the mean ± SEM (n = 3). **C** The TUNEL assay was performed to detect cell apoptosis. FITC staining (green) marks DNA fragmentation. Significance levels are indicated as follows: ns, not significant; *p < 0.05, **p < 0.01, #p < 0.05, ##p < 0.01, &p < 0.05, &&p < 0.01

To study the sepsis model at the cellular level, we first determined the optimal stimulatory concentration of LPS (Fig. 5B). To confirm the extent of LPS-induced damage to DC2.4 cells, we detected cell viability, autophagosome expression, and the expression of apoptosis-related genes Bcl-2 and Bax and performed the TUNEL assay to detect apoptosis. We then selected 5 µg/mL LPS as the optimal LPS concentration to treat DC2.4 cells according to the cell viability assay. The viability of LPS-treated DC2.4 cells decreased, but IFNγ treatment promoted the recovery of cell viability; administration of 2-DG significantly reduced the IFNγ-induced recovery of cell viability. Both 2-DG and LY294002 inhibit the therapeutic effect of IFNγ; however, 2-DG completely prevent the therapeutic effect of IFNγ (Fig. 5B). In order to evaluate the LPS-induced sepsis cell damage from different perspectives, we also examined the autophagy and apoptosis of cells. LC3 puncta were used to label the autophagosomes. According to our image analysis, the expression of autophagosomes in the LPS-induced sepsis group increased and that IFNγ treatment reduced the expression of autophagosomes. Both 2-DG and LY294002 significant inhibit the effect of IFNγ on CLP treatment (Fig. 6A). Next, we determined the expression of Bcl-2 and Bax, which are anti- and pro-apoptotic genes, respectively, and TUNEL detection to study the apoptosis-related changes in each group. Bcl-2 can prevent the apoptosis of some cells (such as lymphocytes), and Bax can bind to and antagonise the apoptosis inhibitor Bcl-2 to accelerate programmed cell death. The expression of apoptotic genes was different from the previous indicators: LPS-induced sepsis decreased Bcl-2 expression, increased Bax expression, and increased apoptosis. Therefore, IFNγ has an inhibitory effect on LPS-induced sepsis-related cell apoptosis. However, the effect of 2-DG was different. Treatment with 2-DG aggravated the decrease in Bcl-2 expression. Unlike the presumed increase in Bax expression, 2-DG also inhibited Bax expression. The same phenomenon was also found in some studies (Zheng et al. 2017a). The results of the TUNEL assay were similar to those of the autophagy assay. LPS induced DC apoptosis, while IFNγ treatment inhibited DC apoptosis; 2-DG and LY294002 inhibited the therapeutic effect of IFNγ (Fig. 6B). In order to clarify the role of IFNγ in the apoptosis of immune cells during sepsis, apoptosis was quantitatively detected via the TUNEL assay. The results showed that IFNγ inhibit the apoptosis caused by sepsis and that 2-DG aggravated this effect. Hence, the therapeutic effect of IFNγ be inhibited (Fig. 6C).

### CLP and LPS-induced sepsis can cause metabolic paralysis; IFNγ restores metabolism by promoting the Warburg effect

**Fig. 7:**
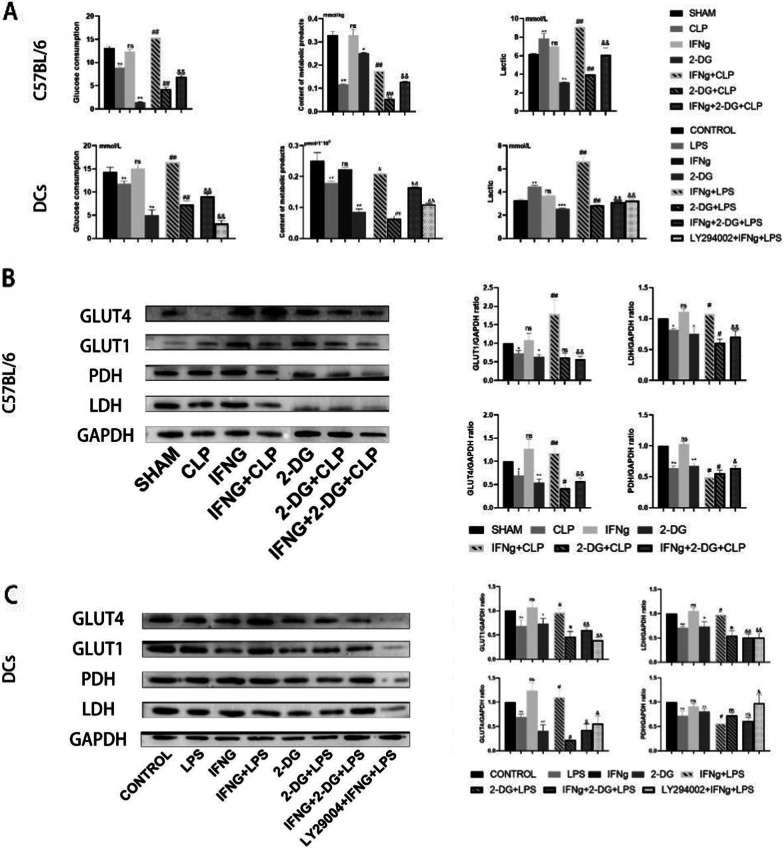
**A** Detection of glucose consumption, extracellular lactate production, and intracellular ATP content. Data are expressed as the mean ± SEM (n = 6). **B** Western blot detection of Warburg effect-related protein expression in the animal models. **C** Western blot detection of Warburg effect-related protein expression in the cell models. Data are expressed as the mean ± SEM (n = 3). Significance levels are indicated as follows: ns, not significant; *p < 0.05, **p < 0.01, #p < 0.05, ##p < 0.01, &p < 0.05, &&p < 0.01

In order to explore the effects of sepsis, IFNγ, 2-DG, and LY294002 on metabolism, commercially available kits were used to detect glucose uptake and the content of extracellular lactate, intracellular ATP, and metabolism-related proteins. Increased glucose uptake, increased extracellular lactate, and decreased intracellular ATP content promote the Warburg effect, The opposite result is that the effect is suppressed. From our animal models and metabolic studies of DC2.4 cells, it can be seen that the immunosuppressive stage of sepsis lead to defects in energy metabolism. Specifically, the sepsis group had reduced glucose uptake, increased extracellular lactate, and decreased intracellular ATP content, which are manifestations of energy metabolism deficits (Fig. 7A). IFNγ treatment promote the Warburg effect and restore metabolism. In contrast, 2-DG inhibit the Warburg effect; specifically, 2-DG and LY294002 inhibit the ability of IFNγ to restore the Warburg effect.

The expression of Warburg effect-related proteins PDH, LDH, GLUT1, and GLUT4 were detected using western blot and immunohistochemistry. LDH, GLUT1, and GLUT4 are glycolysis-related proteins, while PDH is an oxidative phosphorylation-related protein. The increase in the expression of glycolysis-related proteins and the decrease in PDH expression are manifestations of the promotion of the Warburg effect. Conversely, the inverse phenomena indicate the inhibition of the Warburg effect. From the results, the expressions of all Warburg effect-related proteins in the CLP- and LPS-induced sepsis groups were decreased, proving that metabolism was inhibited. Treatment with 2-DG and LY294002 significant inhibit the recovery of metabolic capacity elicited by IFNγ treatment (Fig. 7B, C).

### IFNγ regulates sepsis-induced immunosuppression by promoting the Warburg effect through the PI3K/AKT/mTOR pathway

**Fig. 8:**
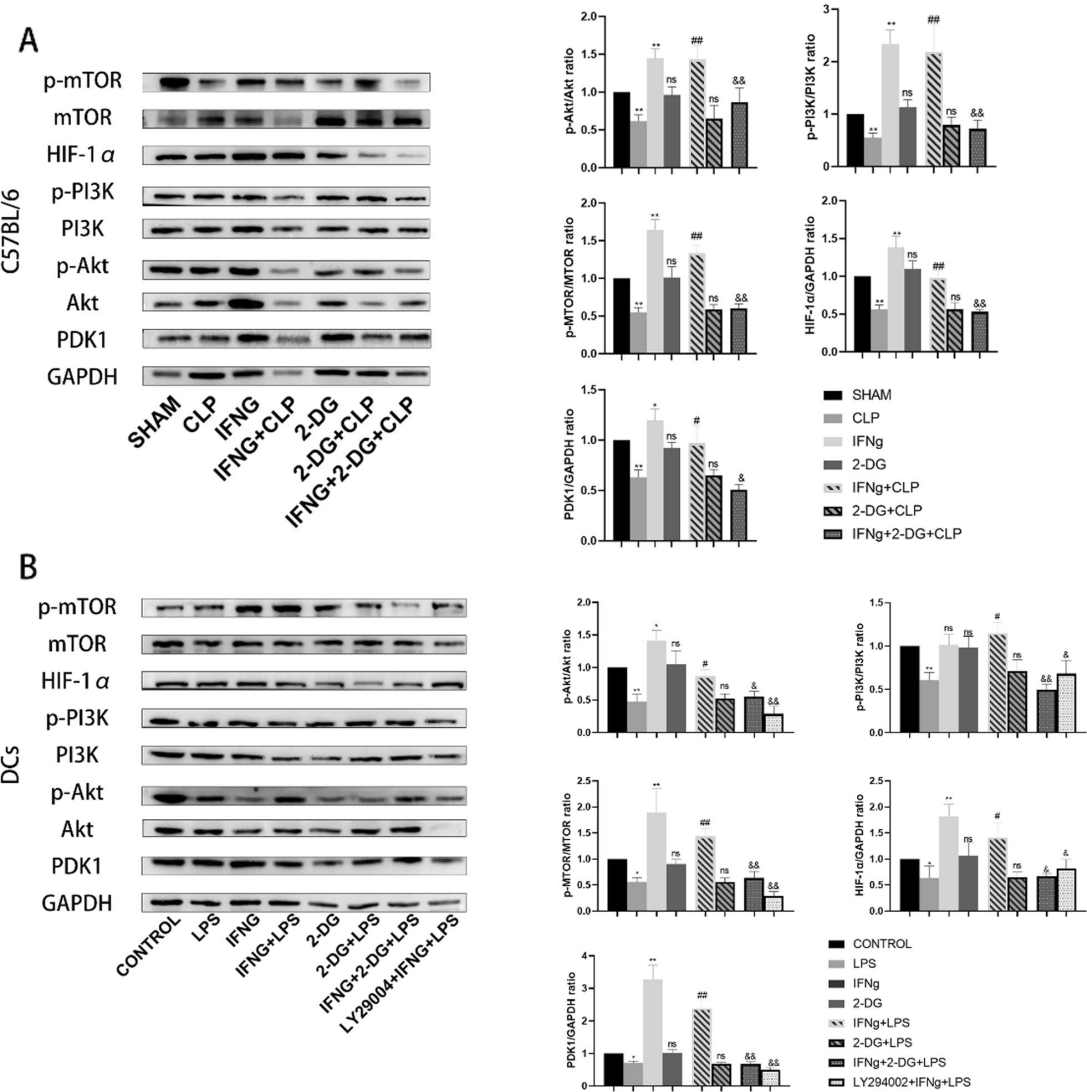
**A** Western blot detection of the expression of p-PI3K/PI3K, p-AKT/AKT, p-MTOR/MTOR, HIF-1α, and PDK1 pathways in the animal models. **B** Western blot detection of the expression of p-PI3K/PI3K, p-AKT/AKT, p-MTOR/MTOR, HIF-1α, and PDK1 pathways in the cell model. Data are presented as the mean ± SEM (n = 3). Significance levels are indicated as follows: ns, not significant; *p < 0.05, **p < 0.01, #p < 0.05, ##p < 0.01, &p < 0.05, &&p < 0.01

The mechanism of the Warburg effect involves the activation of AKT through PI3K phosphorylation. Activated AKT then upregulates the expression of HIF-1α through mTOR. HIF-1α upregulates the expression of pyruvate dehydrogenase kinase 1 (PDK1), a key regulatory enzyme in glucose metabolism. PDK1 inhibits PDH activity and promotes the conversion of glucose metabolism to the glycolysis pathway under aerobic conditions. It is speculated that IFNγ also reverses sepsis-induced immunosuppression through the PI3K/AKT/mTOR/HIF-1α pathway. To verify this hypothesis, a western blot was used to detect the expression of the PI3K/AKT/mTOR pathway and related proteins. In addition, the Warburg effect inhibitor 2-DG and PI3K inhibitor LY294002 were used to verify whether IFNγ in improving sepsis-induced immunosuppression through this pathway. After normalising the grey values, the expressions of p-PI3K/PI3K, p-AKT/AKT, p-MTOR/MTOR, HIF-1α, and PDK1 were decreased in CLP and LPS-induced sepsis, and the Warburg effector pathway was inhibited. IFNγ can promote the expression of this pathway; however, 2-DG did not inhibit the expression of this pathway. Nonetheless, 2-DG significant inhibit the restorative effect of IFNγ on this pathway. The therapeutic effect of IFNγ was significantly inhibited after LY294002 inhibited PI3K pathway. These results are sufficient to prove that IFNγ plays a therapeutic role in sepsis through this pathway and that blocking this pathway inhibit the therapeutic effect of IFNγ (Fig. 8A, B).

In summary, this study investigated the damage induced by sepsis to the body and cells, the inhibition of the immune system, and the impact of sepsis on metabolism in vivo and in vitro. Our results also demonstrated that IFNγ treats sepsis by promoting the Warburg effect through the PI3K/AKT/mTOR pathway by reducing the damage, immunosuppression, and metabolic paralysis brought about by sepsis.

## Discussion

Curing sepsis remains a difficult problem. The general emphasis is on the role of immunosuppression in the development of sepsis, as it is believed that immunosuppression may be the cause of high mortality in sepsis. Therefore, it is important to find a way to treat sepsis-related immunosuppression. There are many available immunotherapeutic methods for sepsis. IFNγ activates effector immune cells and enhances antigen presentation. Our previous experiments also verified this, but its treatment mechanism remains unclear (Wang et al. 2017). This study demonstrated that animal models of CLP lead to immunosuppression, characterised by decreased lymphocyte cytokine secretion capacity, increased Treg percentage, decreased HLA-DR expression, decreased DC presentation, and increased negative regulation of lymphocytes. LPS-induced sepsis promotes apoptosis and autophagy.

This study found that leucocytes in the sepsis tolerance stage have impaired immune and metabolic functions. The metabolic impact of sepsis is comprehensive and has been shown to result in reduced oxygen consumption and fatty acid transport (Cheng et al. 2016). Our study more comprehensively demonstrates the inhibitory effect of sepsis on metabolism in terms of glucose uptake, intracellular ATP production, and extracellular lactate production. Metabolic disorders and mitochondrial damage are thought to be associated with sepsis survival and organ dysfunction, and that elevated intracellular ATP in patients with sepsis predicts better survival (Carré et al. 2010; Carré and Singer 2008). To investigate the role of glycolysis during the immunosuppressive phase of sepsis, we used 2-DG, which acts on hexokinase (the rate-limiting step of glycolysis) to inhibit glycolysis.Aerobic glycolysis is activated during the acute inflammatory phase of sepsis, and the development of sepsis prompts regulatory pathways to suppress host defence mechanisms when metabolism, including glycolysis, is inhibited (Belikova et al. 2007). Restoring the metabolic function of leucocytes in the tolerance phase has become an immunotherapeutic target in sepsis. All studies on the warburg effect in sepsis now focus on the hyperinflammatory phase, reducing energy production by inhibiting the Warburg effect and suppressing the inflammatory storm caused by overactivation of the immune system. By the use of drugs such as 2-DG it was found that suppression of the early inflammatory storm was beneficial to the early survival rate of septic mice, the development of excessive inflammation into exhaustion leads to immune system suppression, and the application of 2-DG at this stage exacerbates the immune suppression of sepsis, and from our survival curves it was found that early 2-DG improved the survival rate, but the continued suppressive effect decreased the survival rate. There is no clear separation marker between the hyperinflammatory and immunosuppressive phases of sepsis, and it has also been suggested in the literature that hyperinflammation and immunosuppression may co-exist in vivo, so caution should be exercised regarding the use of 2-DG in the treatment of sepsis (Lin et al. 2018).

IFNγ is a known sepsis immunostimulatory drug that can improve metabolism. IFNγ enhances antigen presentation, phagocytosis, and cytotoxic functions by acting on antigen-presenting cells (Burke and Young 2019). The role of IFNγ in the immunosuppressive therapy of sepsis has been validated in clinical practice. Adjuvant immunotherapy with IFNγ was well-tolerated, improving immune function in sepsis-induced immunosuppression (Payen et al. 2019; Delsing et al. 2014; Nalos et al. 2012). Studies have also demonstrated that IFNγ modulates DC metabolism by converting oxidative phosphorylation to glycolysis through the mTOR/HIF-1α pathway (increasing glycolysis in the cytosol for lactic acid fermentation, not in the mitochondria, through low-rate glycolysis), and restore glycolysis in sepsis-tolerant leucocytes (Cheng et al. 2016; Pantel et al. 2014). mTOR is a key checkpoint in immune cell metabolism in sepsis. mTOR activation is completely inhibited in CD4^+^ lymphocytes of patients with sepsis; however, AMPKα activation was only slightly affected, and in the case of mTOR blockade, stimulated T cells were unresponsive (Venet et al. 2017; Powell et al. 2012; Delgoffe and Powell 2015).

The HIF-1α pathway and glycolysis are integral to the immune response to sepsis. Innate immune cells lack myeloid-specific HIF-1α, resulting in an inability to produce β-glucan-induced protection against Staphylococcus aureus-induced sepsis (Cheng et al. 2014b). Furthermore, the activation of mTOR by insulin or colony-stimulating factor was found to be mediated by the intermediate activation of the AKT/PI3K pathway (Kelley et al. 1999; Pan et al. 2022b). To this end, our study enriches the mechanism of IFNγ in the treatment of sepsis-induced immunosuppression and proves that IFNγ restores leucocyte metabolic disorder through the PI3K/AKT/mTOR/HIF-1α pathway, thereby restoring the function of immune cells and rebuilding the host immune defence. mTOR promotes anabolic processes to stimulate cell growth, while AMPK promotes the expression of metabolic pathways to maximise energy production. However, the activation of AMPK inhibits the mTOR pathway (MacIver et al. 2013). The restorative effects of IFNγ on metabolism also lead to the increased production of lactate, an alternative energy source for T cells, which stimulates cytokine production in activated and differentiated T cells (Slack et al. 2015; Fischer et al. 2007). However, the accumulation of lactate impairs the proliferation of CD4^+^ T cells. Elevated blood lactate concentration (hyperlactatemia) is a hallmark of septic shock, and lactate levels are positively correlated with disease severity, morbidity, and mortality in sepsis. Hence, the use of IFNγ should be timely and appropriate (Suetrong and Walley 2016; Rishu et al. 2013). IFNγ differentiates macrophages into the M1 phenotype, which is associated with increased glycolysis; blocking glycolysis by 2-DG inhibits IFNγ-induced macrophage differentiation and activation (Wang et al. 2018). Interestingly, IFNg treats sepsis via the P pathway, and 2-DG inhibits the therapeutic effect of IFNg, but 2-DG has no significant inhibitory effect on PI3K passage, which could potentially imply the existence of other important pathways for IFNg to promote the Warburg effect,a possible speculation is that the RAS/RAF/MEK/ERK/cMyc pathway(Sun et al. 2016; Vaupel and Multhoff 2021).

DCs are susceptible to sepsis-induced apoptosis, with reduced HLA-DR expression, elevated IL-10 secretion, and induction of T cell anergy or Treg cell proliferation (Hotchkiss et al. 2002). Decreased HLA-DR expression is a surrogate marker of monocyte anergy, development of persistent infection, patient mortality, and decreased pro-inflammatory cytokine secretion, consistent with the results obtained in our study (Cazalis et al. 2013). Here, we investigated the reduction in the number of DCs induced by sepsis from multiple perspectives of apoptotic gene expression, DNA fragmentation, and autophagy. IFNγ-induced metabolic restoration also restored the number of DCs. Coupled with the promotion of CD86 expression on the surface of primary DCs, metabolic restoration is beneficial to the number and immune function of DCs. Blocking IFNγ and metabolic recovery aggravated apoptosis and autophagy. Studies have demonstrated that DC-related sepsis survival is associated with the expression of co-stimulatory molecules CD80 and CD86 (Benjamim et al. 2005). Both LPS and IFNγ promoted PD-L1 expression, while 2-DG decreased PD-L1 expression, indicating that PD-L1 is not a reliable marker of immunosuppression. One study found that PD-L1 expression was elevated, but CD4^+^ and CD8^+^ T cell activation was not reduced by IFNγ treatment. Preventing sepsis-induced DC apoptosis or enhancing the immune function of DCs can improve the long-term survival rate in sepsis; moreover, these are also the goals of future sepsis therapeutic interventions (Pastille et al. 2011).

There is a relationship between metabolism and cytokine production (Martin et al. 2009), and our study confirmed this. After IFNγ restored metabolism, the ability of lymphocytes to secrete cytokines was also restored. If the therapeutic effect of IFNγ was hindered, the ability of lymphocytes to secrete cytokines could not be restored. IL-10 is a marker of compensatory anti-inflammatory pathways. The results showed that IFNγ had no effect on the ability of septic lymphocytes to secrete IL-10, but treatment with 2-DG inhibited the ability of these cells to secrete IL-10. Therefore, IFNγ can selectively promote the expression of TNF-α and IL-6 without affecting the secretion of the negative regulator IL-10.

IFNγ could also promote Bcl-2 and inhibit Bax expression in sepsis. However, 2-DG inhibited the expression of Bcl-2 and Bax during sepsis; LY294002 did not affect Bax expression even after IFNγ treatment. In alveolar rhabdomyosarcoma, 2-DG-induced cell death is associated with its ability to activate Bax and Bak (Ramirez-Peinado et al. 2011). However, in sepsis-associated cardiac dysfunction, 2-DG administration inhibits the sepsis-induced expression of apoptosis inducers Bax and Bak, as well as JNK phosphorylation in the myocardium (Zheng et al. 2017b). These contrasting results imply that the relationship of 2-DG to apoptosis inducers Bax and Bak requires further investigation.

Sepsis induces the Warburg effect in monocytes to respond to invading pathogens robustly and rapidly by producing pro-inflammatory cytokines and enhancing their phagocytic capacity (Zinkernagel et al. 2008). The therapeutic effect of IFNγ (such as increased cytokine production capacity and increased expression of CD86 on the surface of DCs) was consistent with the Warburg effect. Most research on the Warburg effect and sepsis focused on overcoming the Warburg effect and reducing the expression of HMGB1 (high mobility group protein) by agents including celastrol, shikonin, and capsaicin, which inhibit the PKM2-mediated Warburg effect. The epidermal growth factor receptor inhibitor erlotinib also effectively attenuates CD4^+^ T lymphocyte depletion by downregulating the Warburg effect during sepsis, which promotes mitochondrial damage induced by uncoupling protein 2 in septic acute kidney injury (Yang et al. 2014; Luo et al. 2022; Zhang, et al. 2022; Ji et al. 2021; Huang 2022). Unlike the large body of research associating the Warburg effect and sepsis, our study complements the relationship between the Warburg effect and immunotherapy. For patients in the immune paralysis stage of sepsis, re-inhibition of the Warburg effect is not conducive to their immune function; it even aggravates the damage to their immune system and organ function. Our experiments comprehensively demonstrated the immune stimulation mechanism of IFNγ from two parts in vivo and in vitro, and found that sepsis immunosuppression can be treated by modulating the Warburg effect. In addition, IFNγ can regulate the differentiation of macrophages, and the relationship between the regulatory mechanism and the Warburg effect needs further study. The Akt-mTOR-HIF-1α signaling pathway and glycolytic-related enzymes are both targets for intervention in the Warburg effect, which open up new avenues for immunosuppressive therapy for sepsis. To evaluate the effect of IFNγ against specific phases of immune response and to more realistically simulate the clinical sepsis patient setting, new studies could use either a two-step CLP model (CLP followed by Streptococcus pneumoniae induced pneumonia) or an endotoxin tolerance model (LPS pretreatment followed by establishment of CLP), but to address the high mortality rate of the two-step CLP model (Muenzer et al. 2006). The mode of administration of IFNg should also be optimized. Administration 30 min in advance is not consistent with the clinical treatment of sepsis, and it is difficult to predict the onset of sepsis in advance in the clinic. Administration after the sepsis model is established, or until immunosuppression occurs can better simulate the clinical environment.Moreover, for the different states of the specific immune cell Warburg effect, regulating the metabolism of immune cells characterized by the Warburg effect can achieve individualized intervention in sepsis treatment.

## Conclusions

Immunosuppression caused by sepsis is fatal in humans, and we found that the cause of sepsis immunosuppression is inseparable from metabolic paralysis. IFNγ is a clinically proven sepsis immunotherapy whose effect is inseparable from metabolic regulation. By investigating the mechanisms underlying metabolic inhibition and the PI3K/AKT/mTOR pathway, it was confirmed that IFNγ promotes the Warburg effect through the PI3K/AKT/MTOR pathway to treat immunosuppression in septic mice. Hence, the mechanism of action of IFNγ was elucidated, providing a new target for the immunotherapy of sepsis.

## Data Availability

All data generated or analysed during this study are included in this published article.
