# Correction to: Interferon-γ regulates immunosuppression in septic mice by promoting the warburg effect through the PI3K/AKT/mTOR pathway

**DOI:** 10.1186/s10020-023-00710-w

**Published:** 2023-08-17

**Authors:** Xu-zhe Fu, Yu Wang

**Affiliations:** Department of Emergency Medicine, Shengjing Hospital of China Medical University, Shenyang, China

**Correction: Molecular Medicine (2023) 29:95**10.1186/s10020-023-00690-x.

Following publication of the original article [1], the authors informed us that the affiliation department was incorrectly added.

The authors affiliation has been updated above and original article has been corrected.
